# Betaine Aldehyde Dehydrogenase (*BADH*) vs. Flavodoxin (*Fld*): Two Important Genes for Enhancing Plants Stress Tolerance and Productivity

**DOI:** 10.3389/fpls.2021.650215

**Published:** 2021-04-01

**Authors:** Mohsen Niazian, Seyed Ahmad Sadat-Noori, Masoud Tohidfar, Seyed Mohammad Mahdi Mortazavian, Paolo Sabbatini

**Affiliations:** ^1^Field and Horticultural Crops Research Department, Kurdistan Agricultural and Natural Resources Research and Education Center, Agricultural Research, Education and Extension Organization (AREEO), Sanandaj, Iran; ^2^Department of Agronomy and Plant Breeding Science, College of Aburaihan, University of Tehran, Tehran, Iran; ^3^Department of Plant Biotechnology, Faculty of Sciences & Biotechnology, Shahid Beheshti University, G.C., Tehran, Iran; ^4^Department of Horticulture, Michigan State University, East Lansing, MI, United States

**Keywords:** compatible solutes, flavoproteins, glycine betaine, osmotic adjustment, photosynthetic electron transport chain, stress

## Abstract

Abiotic stresses, mainly salinity and drought, are the most important environmental threats that constrain worldwide food security by hampering plant growth and productivity. Plants cope with the adverse effects of these stresses by implementing a series of morpho-physio-biochemical adaptation mechanisms. Accumulating effective osmo-protectants, such as proline and glycine betaine (GB), is one of the important plant stress tolerance strategies. These osmolytes can trigger plant stress tolerance mechanisms, which include stress signal transduction, activating resistance genes, increasing levels of enzymatic and non-enzymatic antioxidants, protecting cell osmotic pressure, enhancing cell membrane integrity, as well as protecting their photosynthetic apparatus, especially the photosystem II (PSII) complex. Genetic engineering, as one of the most important plant biotechnology methods, helps to expedite the development of stress-tolerant plants by introducing the key tolerance genes involved in the biosynthetic pathways of osmolytes into plants. Betaine aldehyde dehydrogenase (*BADH*) is one of the important genes involved in the biosynthetic pathway of GB, and its introduction has led to an increased tolerance to a variety of abiotic stresses in different plant species. Replacing down-regulated ferredoxin at the acceptor side of photosystem I (PSI) with its isofunctional counterpart electron carrier (flavodoxin) is another applicable strategy to strengthen the photosynthetic apparatus of plants under stressful conditions. Heterologous expression of microbially-sourced flavodoxin (*Fld*) in higher plants compensates for the deficiency of ferredoxin expression and enhances their stress tolerance. *BADH* and *Fld* are multifunctional transgenes that increase the stress tolerance of different plant species and maintain their production under stressful situations by protecting and enhancing their photosynthetic apparatus. In addition to increasing stress tolerance, both *BADH* and *Fld* genes can improve the productivity, symbiotic performance, and longevity of plants. Because of the multigenic and complex nature of abiotic stresses, the concomitant delivery of *BADH* and *Fld* transgenes can lead to more satisfying results in desired plants, as these two genes enhance plant stress tolerance through different mechanisms, and their cumulative effect can be much more beneficial than their individual ones. The importance of *BADH* and *Fld* genes in enhancing plant productivity under stress conditions has been discussed in detail in the present review.

## Introduction

Drought and salinity are the most important abiotic stresses that are currently challenging global food security by reducing agricultural productivity and quality. The ever-increasing population of the world needs constant improvement in crop productivity to meet the growing food demands. Salinity stress is more pronounced in agricultural regions with water scarcity and inadequate irrigation (Isayenkov, 2019). Cellular dehydration is inevitable in salinity stress, which makes the mechanism of drought and salinity stress similar (Shailani et al., 2020; Wai et al., 2020). Therefore, increasing tolerance to salinity and drought stress can be done in similar ways. Drought and salinity impair the photosynthesis and transpiration of plants by decreasing levels of chlorophyll and carotenoids, distorting the chloroplast ultrastructure and photosystem II (PSII) complex, and reducing stomatal conductance (Pan et al., 2020). Photosynthesis is a pivotal physio-biochemical process of plants that is also highly sensitive to abiotic stresses. Abiotic stresses have adverse effects on PSII activity by disintegrating PSII reaction centers, oxygen-evolving complex (OEC), and limiting the activity of quinone acceptors. The photosynthetic electron transport chain (PETC) in plants, especially electron carriers at the acceptor side of photosystem I (PSI), are vulnerable to abiotic stresses (Arif et al., 2020; Chen et al., 2020). Cellular dehydration and toxicity along with high accumulation of reactive oxygen species (ROS) are other consequences of drought and salinity, which eventually lead to programmed cell death (PCD) (Latif et al., 2020). Although, reactive oxygen species oxidize and disrupt some cellular components compromising their original functions, at low levels, they can act as important signaling molecules and second messengers to regulate plant growth and stress responses. Therefore, maintaining the equilibrium of production and the scavenging of ROS is important; abiotic stresses disturb this balance, leading to increased intracellular ROS levels (Huang et al., 2019). Plants respond to harsh environments by applying an array of tolerance mechanisms. Cellular detoxification activation, osmoprotectants accumulation, antioxidant machinery activation (both enzymatic and non-enzymatic), signaling pathways activation/regulation, and ROS scavenging are involved in enhancing plant stress tolerance (Niazian and Shariatpanahi, 2020; Shailani et al., 2020). Cell osmotic pressure adjustment and the protection of cell membranes and photosynthesis machinery coupled with other functional macromolecules are vital measures to eliminate the harmful effects of abiotic stresses (Niazian et al., 2019). The activation of biosynthetic enzymes to produce higher levels of stress protectants and antioxidant compounds is one of the plant's strategies to quench the harmful oxidizing molecules (Wai et al., 2020).

Genetic engineering is one of the widely applied biotechnology-based breeding methods (BBBMs) to enhance a plant's resistance to stressful conditions. Key tolerance genes, explored from different sources, have been introduced into various plant species to improve abiotic stress tolerance. According to the functional analysis of gene products, the genes of interest for enhancing abiotic stresses tolerance are involved in (i) encoding ion transporters, (ii) osmolyte biosynthesis, (iii) antioxidant machinery, (iv) encoding stress-induced proteins, and (v) encoding transcription factors (Isayenkov, 2019). All of these genes try to mitigate the adverse effects of abiotic stresses and keep the photosynthesis efficiency and subsequent productivity of plants at an acceptable level. The introduction of *K2-NhaD*, a plasma membrane Na^+^/H^+^ antiporter encoding gene, to upland cotton (*Gossypium hirsutum* L.), led to a considerable increase of relative water content, chlorophyll, soluble sugar, proline levels, and superoxide dismutase (SOD), catalase (CAT), and peroxidase (POX) activity in transgenic plants more so than wild types under drought and salinity conditions (Guo et al., 2020). The overexpression of the *IbCBF3* transcription factor enhanced low temperature and drought stress tolerance in transgenic sweet potatoes (*Ipomoea batatas* [L.] Lam) (Jin et al., 2017). Zhou et al. (2020a) transferred the *Arabidopsis* malonyl-CoA synthetase gene AAE13.1 (*AtAAE13.1*) to cell suspension cultures of rice (*Oryza sativa* L.), cotton (*Gossypium hirsutum* L.), and white pine (*Pinus strobus* L.) using the GV3101 strain of *Agrobacterium tumefaciens*. Reportedly, salinity stress tolerance was enhanced in transgenic cells with higher cell viability and growth rates and increased content levels of amino acids, glycolate, phosphoglycolate, sucrose, glucose, and fructose. It also showed lower lipid peroxidation and an increased oxidation rate (Zhou et al., 2020a). Overexpression of the *NtabDOG1L* gene showed drought tolerance improvement in tobacco (*Nicotiana tabacum*) by increasing antioxidant capacity and up-regulating the expression of drought defense genes (Zhang X. et al., 2019). There are some other key genes that can improve plant stress tolerance through a combination of the above-mentioned functions. In rice, overexpression of the *OsRab7* gene enhanced drought and heat tolerance by modulating osmolytes, antioxidants, and abiotic stress-responsive gene expression (El-Esawi and Alayafi, 2019a). Overexpression of the *StDREB2* transcription factor increased drought tolerance in cotton by improving gas-exchange performance, ROS scavenging, antioxidant enzyme activities, osmolytes accumulation, and expression of stress-related genes (El-Esawi and Alayafi, 2019b). The introduction of the *codA* gene from *Arthrobacter globiformis* into the potato (*Solanum tuberosum*) plastid genome, led to higher glycine betaine (GB) osmolyte accumulation, and higher relative water and chlorophyll content of the transformed plants grown under drought stress conditions (You et al., 2019).

**Figure 1:**
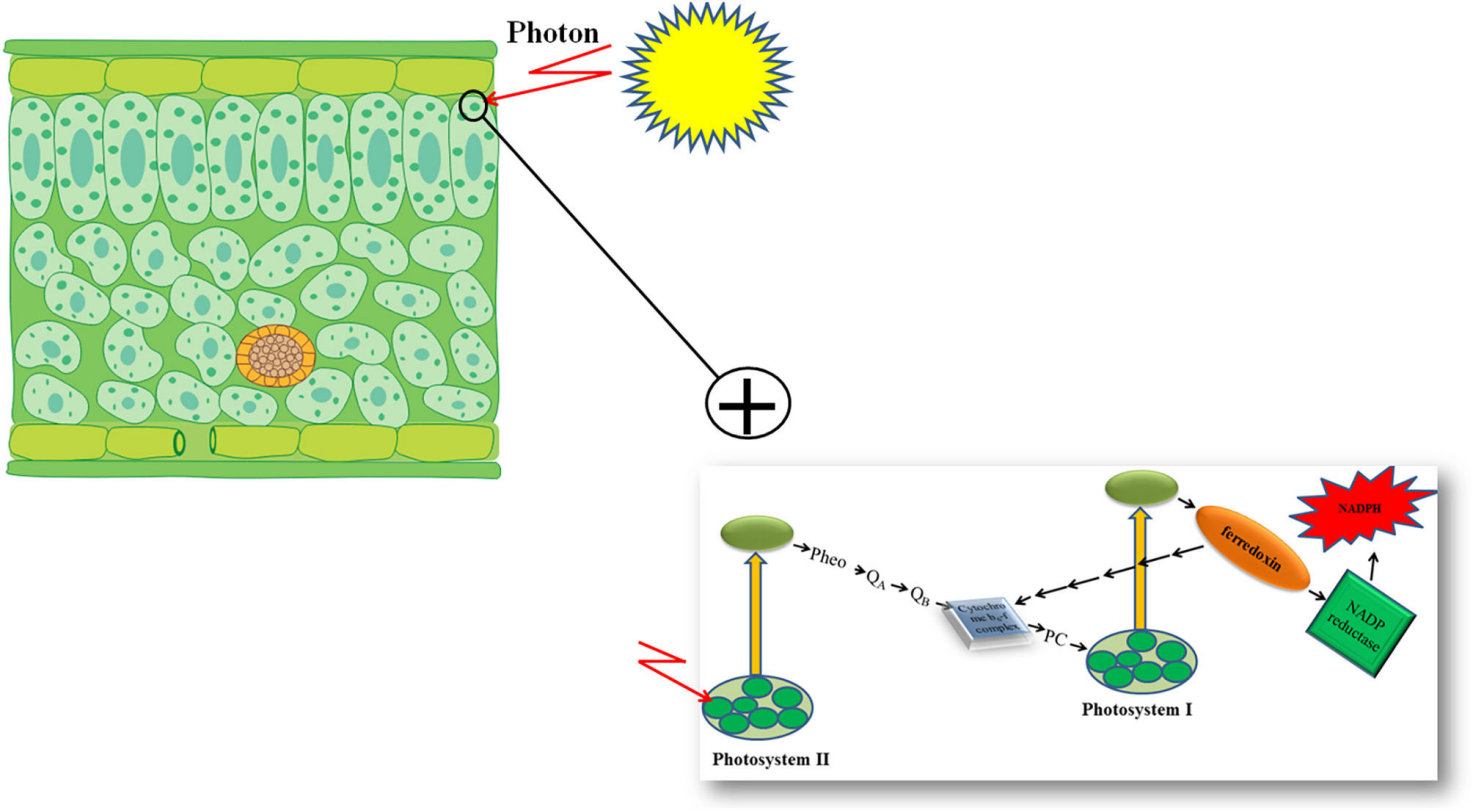
Schematic presentation of photosynthetic electron transport chain of plants.

Furthermore, genetic engineering increased the photosynthetic efficiency of plants under stressful conditions via the substitution of a stress-vulnerable key electron carrier at the acceptor side of PSI in the PETC (Zurbriggen et al., 2010). Ferredoxin (Fd)—the final electron acceptor of the PETC—is a multifunctional electron carrier iron-sulfur protein in photosynthetic organisms. Ferredoxin has critical roles in the CO_2_ fixation cycle, nitrogen/sulfur assimilation, amino acid synthesis, fatty acid desaturation, antioxidant regeneration, and different regulation, dissipation, and biosynthetic pathways (Zurbriggen et al., 2008; Gharechahi et al., 2015). Through interaction with Fd–NADP^+^ reductase (FNR), photoreduced Fd can generate the NADPH required for carbon assimilation and other biosynthetic and protective pathways (Figure 1). In addition, Fd can affect different metabolic regulatory and dissipative routes of the stroma by diverting reducing equivalents back to the cytochrome b_6_f complex in the PETC or to soluble electron acceptor enzymes involved in the pathways (Figure 1) (Giró et al., 2011). The significant role of Fd in plant stress responses has also been reported in several studies (Ma et al., 2008). However, environmental stresses can significantly down-regulate Fd expression and NADP^+^ deficiency and therefore compromise plant survival. Photosynthetic microorganisms like cyanobacteria and some algae have an alternative strategy in stressful environmental conditions. They compensate for a deficiency of Fd expression by activating the expression of flavodoxin (Fld)—an isofunctional mobile electron carrier protein that contains a prosthetic group of flavin mononucleotide—and keep their photosynthetic efficiency under stressful situations like iron deficiency, high illumination, salinity, and drought (Zurbriggen et al., 2007).

Increasing the accumulation of protective compatible solutes with compensation for a reduced level of Fd in PETC, through genetic engineering techniques, are two fast and applicable strategies to enhance stress tolerance of plants and to maintain their photosynthetic efficiency and productivity under stressful conditions. The principles of increasing plant stress tolerance using these two strategies are presented in the following sections of the present review.

## Compatible Solutes and Osmotic Adjustment

Osmotic tolerance is one of the first plant responses to challenging environmental conditions, and it is achieved through rapid long-distance (root to shoot) signaling mechanisms (Isayenkov and Maathuis, 2019). Mobile molecules, such as small proteins, peptides, RNAs, metabolites, and second messengers, are involved in long-distance signaling in plant stress response (Takahashi and Shinozaki, 2019). Osmoprotectants/compatible solutes, including amino acids (proline), betaines (glycine betain), polyamines, and sugars are low-molecular-weight highly soluble non-toxic compounds. They act as ROS scavengers and are involved in a wide range of biological processes through interaction with antioxidative enzymes, stabilizing proteins, and plasma membranes (Chen and Murata, 2002). Despite their different biochemical groups, compatible solutes have similar roles in protecting plants against stresses (Surabhi and Rout, 2020). These organic osmolytes have significant roles in enzyme and membrane integrity and in the osmotic adjustment of plants under stress conditions (Ashraf and Foolad, 2007). A comparative analysis of 11 sugar beet cultivars under drought stress reported that drought-tolerant cultivars increased the accumulation of proline and GB osmolytes (Islam et al., 2020). Exogenous proline significantly enhanced cell membrane integrity, leaf water content, and photosynthetic efficiency and also up-regulated the osmoprotectant content of onions (*Allium cepa* L.) under drought stress growing conditions (Semida et al., 2020).

Osmolytes and related enzymes in their metabolism are important in plant survival under abiotic stress (Ozturk et al., 2020). Enhancing the endogenous accumulation of organic solutes responsible for cellular osmotic adjustment, such as mannitol, glycine, betaine, proline, myo-inositol, and sugars, by up- or down-regulation of genes involved in their synthetic pathway, can increase plants tolerance to major abiotic stresses, especially those of drought and salinity (Paschalidis et al., 2019). Glycine betaine and proline are the most common osmolytes under stress conditions (Ozturk et al., 2020). The exogenous application of proline can be more effective than GB in alleviating the adverse effect of abiotic stresses in some plants (Youssef et al., 2018; Bhuiyan et al., 2019). However, the introduction of GB biosynthetic genes is more effective than other osmoprotectant genes in enhancing plant abiotic stress tolerance (Surabhi and Rout, 2020). Recent studies indicated that the GB is the most important osmoprotectant in inducing stress tolerance (Hasan et al., 2020; Zhou et al., 2020b).

## Glycine Betaine and Betaine Aldehyde Dehydrogenase (*BADH*) Gene

Glycine betaine is one of the main quaternary ammonium compounds (QAC) produced in cash crops. Among prominent betaines in plants, including glycine betaine, proline betaine, and beta alanine, GB is more significant and an extensively studied osmoprotectant. Glycine betaine enrichment is one of the common responses that higher plants have to drought and salinity stresses (Ashraf and Foolad, 2007). Glycine betaine can maintain osmotic adjustment by maintaining a high Na^+^ to K^+^ ratio and lowering the toxic effects of ions through accumulation in the cell (Arif et al., 2020). GB is chemically neutral and can interact with domains of both hydrophilic and hydrophobic macromolecules, such as enzymes and protein complexes, due to its molecular properties (Hasan et al., 2020). By providing osmotic adjustment, GB is able to protect the PSII complex, biomembrane system, and the quaternary structures of other pivotal enzymes and proteins (Golestan Hashemi et al., 2018; Surabhi and Rout, 2020). Betaine is a unique compatible solute, which exists semi-permanently or permanently in plant cells and has slower degradation when compared to other osmolytes (Chen and Murata, 2002). Glycine betaine is able to mitigate the detrimental effects of drought and salinity stresses through a wide range of physio-chemical processes. Through improving the Ca^2+^-ATPase and Hill reaction activities in the thylakoid membrane system, GB increases photosynthesis efficiency under drought stress conditions (Nawaz and Wang, 2020). The foliar application of GB induced the salinity tolerance in onions by up-regulating antioxidative defense systems, including non-enzymatic (glutathione and ascorbic acid), enzymatic [CAT, SOD, and ascorbate peroxidase (APX)] antioxidants, photosynthetic efficiency, and increasing the membrane stability index (Rady et al., 2018). Similarly, the exogenous application of GB enhanced the cold stress tolerance of perennial (*Lolium perenne* L.) and annual (*L. multiflorum*) ryegrass (Mickelbart and Boine, 2020) along with the drought tolerance of sweet potatoes (*Ipomoea batatas*) (Tisarum et al., 2020). A comparative analysis of the exogenous application of abscisic acid (ABA) and GB on enhancing the drought tolerance of *Axonopus compressus* revealed that GB was more effective than ABA. This study showed that exogenous GB application resulted in increasing levels of soluble sugars, proteins, proline, phenolics, and chlorophyll in addition to increasing activity of enzymatic antioxidants SOD, CAT, POX, and APX (Nawaz and Wang, 2020).

Betaine aldehyde dehydrogenase (*BADH*) is one of the genes that encode the enzymes involved in the synthesis of GB and it is responsible for the second oxidation reaction in the GB synthesis pathway. Both choline monooxygenase (*CMO*) and *BADH* genes are important in GB synthesis in higher plants. These two genes can be involved in the transcriptional regulation of different biological processes, such as stress and hormone responses, through their cis-effective regulatory elements (CREs) (Ozturk et al., 2020). Glycine betaine has an important role in manipulating gene expression, as GB-induced expression of specific genes (especially ROS-scavenging enzymes) and transcription factors (Chen and Murata, 2008, 2011; Xu et al., 2018). Yamada et al. (2015) reported that transgenic sugar beet plants with knocked out *CMO*, through antisense *BvCMO*, were more susceptible to salt stress when compared to wild-type plants. These results verified the importance of these two genes in GB synthesis and in enhancing tolerance to abiotic stresses. The *BADH* gene can be cloned from higher plants and other types of living organisms (Chen and Murata, 2002). Spinach (*Spinacia oleracea*), sugar beets (*Beta vulgaris*), barley (*Hordeum vulgare*), quinoa (C*henopodium quinoa*), and rice are the main sources of *BADH* cloning (Yamada et al., 2015; Jiang et al., 2016). Another gene with BADH activity has been reported in *Lycium ruthenicum, LrAMADH1*, which is responsible for increasing GB accumulation under NaCl stress (Liu et al., 2018). Other than abiotic stress mechanisms, multifunctional *BADH* is involved in fragrance production (through polyamines oxidation pathway), and as a selectable marker in the antibiotic-free selection of transgenic plants. This gene is involved in a plant's abiotic stress response through the mitogen-activated protein kinase (MAPK) signaling pathway (Golestan Hashemi et al., 2018). Therefore, *BADH* is involved in a plant's tolerance to abiotic stresses through the MAPK signaling pathway and metabolic synthesis of GB. Introduction of the *BADH* gene can lead to enhanced abiotic stress tolerance in desired plant species. Enhanced tolerance to abiotic stresses in *BADH*-overexpressing transgenic plants has been reported in different plant species (Table 1).

**Table 1:** Examples of transferred *BADH* genes to enhance abiotic stresses in different plants species.

**Plant species**	**Source of delivered *BADH* gene**	**Methods of transformation**	**Targeted stress(s)**	**Improved characteristics**	**References**
Ajowan (*Trachyspermum ammi* L.)	Spinach	*Agrobacterium*-mediated	Drought & salinity	Seedling fresh weight, plant height, proline content, relative water content, secondary metabolites content	Niazian et al., 2019
*Arabidopsis thaliana*	*Ammopiptanthus nanus* Wheat	*Agrobacterium*-mediated *Agrobacterium*-mediated	Drought & Salinity Salinity	Survival rate, fresh weight, relative water content, proline content, relative electrolyte leakage, MDA content Root length, GB content, RELs	Yu et al., 2017; Sun et al., 2019
Chicory (*Cichorium intybus* L.)	Barley	*Agrobacterium*-mediated	Drought & Salinity	K^+^/Na^+^ ratio, GB accumulation, MDA content, chlorophyll content	Li et al., 2016
Maize (*Zea mays* L.)	*Suaeda liaotungensis* kitag-	Pollen-tube pathway	Salinity Drought	GB accumulation, membrane permeability, chlorophyll content Morphological characteristics, GB accumulation, proline content, levels of ROS, CAT, POX, SOD, and MDA	Wu et al., 2008; Zhao et al., 2020
Potato (*Solanum tuberosum*)	*Atriplex canescens*	*Agrobacterium*-mediated	Salinity	Proline and chlorophyll content, H_2_O_2_ and MDA levels	Ali et al., 2020
*Populus nigra L*	-	*Agrobacterium*-mediated	Salinity	Chlorophyll b, SOD activity	Zhou et al., 2020b
Soybeans (*Glycine max*)	*Atriplex canescens*	*Agrobacterium*-mediated	Drought	Germination index, proline content, POX activity, yield components	Qin et al., 2017
Tobacco (*Nicotiana tabacum*)	Spinach	*Agrobacterium*-mediated	High temperature	PSII efficiency, chlorophyll fluorescence induction kinetics, activity of CAT, SOD and APX, ascorbate and glutathione contents	Yang et al., 2007
Tomato (*Solanum lycopersicum*)	Spinach Spinach	*Agrobacterium*-mediated *Agrobacterium*-mediated	High temperature High temperature	Lipid peroxidation, GB accumulation, PSII photochemical activity, hydrogen peroxide, and superoxide anion radical levels GB accumulation, CO_2_ assimilation, PSII photochemical activity, hydrogen peroxide, superoxide anion radical and MDA levels	Li et al., 2014; Zhang et al., 2020
Walnut (*Juglans regia* L.)	Spinach	*Agrobacterium*-mediated	Drought & Salinity	Shoot height, survival rate	Rezaei Qusheh Bolagh et al., 2020
Wheat (Triticum aestivum)	Mountain spinach (Atriplex hortensis L.) Barley	Microprojectile bombardment Agrobacterium-mediated	Salinity Salinity	GB accumulation, chlorophyll and carotenoid contents, photosynthetic efficiency, Ca^2+^-ATPase activity K^+^/Na^+^ ratio, GB accumulation, survival rates	Tian et al., 2017; Li et al., 2019

*CAT, catalase; GB, Glycine betaine; MDA, Malondialdehyde; POX, Peroxidase; PSII, Photosystem II; ROS, Reactive oxygen species; SOD, Superoxide dismutase; REL, Relative electrolytic leakage*.

The *Agrobacterium*-mediated transformation of soybeans by a *BADH* gene from *Atriplex canescens* increased the germination index of transgenic lines under osmotic stress compared with the control plants. In addition, the proline content, POX activity, and yield components of transgenic lines were higher than the control plants (Qin et al., 2017). The *Agrobacterium*-mediated transformation of Persian walnuts (*Juglans regia* L.) *cv*. Chandler using a spinach *BADH* gene led to enhanced tolerance to osmotic (PEG) and salinity (NaCl) stresses (Rezaei Qusheh Bolagh et al., 2020). The overexpression of the *Ammopiptanthus nanus BADH* gene, a xerophyte leguminous shrub, in *Arabidopsis* (*Arabidopsis thaliana*) led to a significantly increased tolerance of *Arabidopsis* transgenic plants to high salt and drought stresses. This was a result of an increased content of GB and proline, higher relative water content, and lower relative electrolyte leakage and malondialdehyde (MDA) content in relation to the untransformed plants (Yu et al., 2017). Li et al. (2019) transferred the *HvBADH1* gene from hulless barley to bread wheat (*Triticum aestivum*) and reported a higher K^+^/Na^+^ ratio, greater accumulation of GB, and higher seedling survival rates of transgenic lines under severe salinity stress (150 mM NaCl). The heterologous expression of the hulless barley *BADH* (*HvBADH1*) gene enhanced drought and salinity stress in chicory (*Cichorium intybus* L.) (Li et al., 2016). Zhang et al. (2020) compared *codA*- and *BADH*-transgenic tomato (*Solanum lycopersicum*) plants under high-temperature stress and reported that GB level, CO_2_ assimilation, and photosystem II (PSII) photochemical activity in transgenic plants were more elevated than in wild types. However, hydrogen peroxide (H_2_O_2_), superoxide anion radical (${\text{O}}_{2}^{-}$), and MDA levels in wild-type plants were all increased than in transgenes ones. Authors also reported that *codA* tomato plants exhibited higher degrees of enhanced thermotolerance than *BADH* tomato plants (Zhang et al., 2020). *BADH* transgenic maize (*Zea mays* L.) plants showed better germination ability, morphological characteristics, higher levels of antioxidant enzymes (CAT, POX, and SOD), and osmotic regulatory substances, as well as a lower accumulation of MDA compared with wild type plants under drought stress conditions (Zhao et al., 2020). Delivery of the *Atriplex canescens BADH* gene to potatoes followed by increased proline and chlorophyll levels as well as decreased H_2_O_2_ and MDA levels in transgenic plants under salinity stress (Ali et al., 2020). Wheat plants transformed with a *BADH* gene from mountain spinach (*Atriplex hortensis* L.) showed higher chlorophyll and carotenoid levels and higher Hill reaction activities as well as higher Ca^2+^-ATPase activity under NaCl stress. The authors also reported that *BADH* transgenes over accumulated GB, which protected the components and functions of their thylakoid membranes followed by their enhanced photosynthesis under salinity stress (Tian et al., 2017). Sun et al. (2019) reported an enhanced GB accumulation and lower relative electrolytic leakage (REL) in *Arabidopsis* plants transformed by a *BADH* gene from salt-tolerant wheat variety DM1 (Dongnongdongmai 1) under salinity stress.

Metabolic engineering of GB, to enhance plant tolerance to abiotic stresses, can be performed by introducing genes from other types of living organisms, like the *codA* gene from *Arthrobacter globiformis* (You et al., 2019), glycine sarcosine methyltransferase (*ApGSMT2*), and dimethylglycine methyltransferase (*ApDMT2*) genes from *Aphanothece halophytica* (Song et al., 2018). A recent study reported overexpression of a novel *GB1* gene that effectively increased the content of GB in transgenic maize and soybean plants (Castiglioni et al., 2018).

The other compatible solutes, besides GB, that accumulated through metabolic engineering include fructan, mannitol, D-Ononitol, proline, sorbitol, and trehalose (Chen and Murata, 2002).

## Flavodoxin Electron Carriers and *FLD* Gene

Flavodoxins are small isofunctional electron carriers with the ubiquitous electron shuttle Fd which shuttles low potential electrons between donors and acceptors in redox-based metabolisms. Flavodoxins are specific to some bacteria and oceanic algae as opposite to ferredoxins, which prevalently distribute in all living organisms. Environmental stresses lead to down-regulation of ferredoxin in photosynthetic microorganisms. However, they make use of flavodoxins to perform Fds functions under challenging environmental conditions. Plants have lost the *Fld* gene and its adaptive advantages during their evolutionary period, somewhere between the green algal ancestor and the first terrestrial plants. Abundance and accessibility of iron in coastal regions and firm land, and the fact that Fd is more efficient than Fld as an electron carrier, are reasonable hypotheses for the loss of the *Fld* gene from their higher plant genetic pool despite its adaptive advantages (Zurbriggen et al., 2007; Pierella Karlusich et al., 2014). On the other hand, studies showed that flavodoxin can compensate for the down-regulation of PETC acceptor Fd. In *Fd* knocked-down transgenic tobacco plants, cyanobacterial *Fld* expression complemented Fd deficiency. Moreover, *Fld* expression restored growth, chlorophyll pigment content, and photosynthetic capacity, and it relieved electron pressure on the electron transport chain (Blanco et al., 2011). The replacement of down-regulated Fd electron acceptor with Fld helped to reconstruct electron delivery to productive routes rather than O_2_ and ROS production (Zurbriggen et al., 2008; Giró et al., 2011; Lodeyro et al., 2012), and subsequently led to a higher degree of stress tolerance. This was first demonstrated in the transgenic tobacco plants with an introduced *Fld* gene, which accumulated lower amounts of ROS under abiotic stress conditions (Tognetti et al., 2006).

Expressing microbially-sourced genes in transgenic plants is a popular strategy for improving plant desired phenotypes (Smith-Moore and Grunden, 2018). *Fld* is one of these microbial genes whose heterologous expression has enhanced plant abiotic stress tolerance. The overexpression of a cyanobacterial *Fld* in creeping bentgrass (*Agrostis stolonifera* L.) enhanced plant performance under oxidative, drought, heat, and nitrogen starvation stresses. It occurred by affecting the expression of heat-shock protein genes, up-regulating the expression of nitrite reductase, and N transporter genes. It also had an impact on increasing production of reduced thioredoxin, water retention, cell membrane integrity, N accumulation, and total chlorophyll content (Li et al., 2017). The comparative proteomic analysis of transgenic tobacco expressing cyanobacterial *Fld* and wild type plants under drought stress conditions, revealed that *Fld* expression enhanced drought tolerance by affecting an abundance of drought-responsive proteins (DRPs) such as remurin, ferredoxin-NADP reductase (FNR), chloroplast manganese stabilizing protein-II, phosphoglycerate mutase, and glutathione S-transferase. These DRPs were involved in carbohydrate and energy metabolism as well as oxidative stress responses (Gharechahi et al., 2015). The heterologous expression of a cyanobacterial *Fld* gene also improved drought and salinity stress tolerance in somatic embryos of Persian walnuts (Sheikh Beig Goharrizi et al., 2016). Transgenic ajowan plants, with an exogenous *Fld* gene introduced from cyanobacterial, showed greater drought tolerance than wild-type plants (Niazian et al., 2017). Transgenic potato plants with an introduced cyanobacterial *Fld* gene in their chloroplasts changed their transcriptional and metabolic profile under drought stress conditions and reported increased photosynthetic activity and consequent tuber yield in addition to a decreased ROS accumulation (Karlusich et al., 2020). A synthesis of *Fld* gene delivery to enhance stress tolerance in different plant species is presented in Table 2.

**Table 2:** Heterologous expression of *Fld* gene to enhance abiotic stresses in different plant species.

**Plant species**	**Source of delivered *Fld* gene**	**Methods of transformation**	**Targeted stress(s)**	**Improved characteristics**	**References**
Ajowan (*Trachyspermum ammi* L.)	Cyanobacterial	*Agrobacterium*-mediated	Drought	Survival rate	Niazian et al., 2017
Canola (*Brassica napus* L.)	–	*Agrobacterium*-mediated	Salinity	–	Nazila et al., 2013
Creeping bentgrass (*Agrostis stolonifera* L.)	Cyanobacterial	*Agrobacterium*-mediated	Drought & Heat & Nitrogen starvation	Water retention, cell membrane integrity, N accumulation, total chlorophyll content	Li et al., 2017
*Medicago truncatula*	*Anabaena* cyanobacterial	*Agrobacterium*-mediated	Salinity	Antioxidant metabolism in nodules, photochemical efficiency of PS II, nitrogen fixation.	Coba de la Peña et al., 2010
Potato (*Solanum tuberosum*)	Anabaena PCC7119 cyanobacterial	Agroinfiltration	Drought	ROS accumulation, photosynthetic activity, tuber yield	Karlusich et al., 2020
Tobacco (*Nicotiana tabacum*)	*Anabaena* cyanobacterial Cyanobacterial Cyanobacterial Anabaena sp.PCC7120 *Anabaena* cyanobacterial	*Agrobacterium*-mediated *Agrobacterium*-mediated *Agrobacterium*-mediated Particle bombardment	Oxidative stress (methyl viologen) & Low temperature & drought & UV radiation Drought Oxidative stress (methyl viologen) Oxidative stress (methyl viologen) & chilling temperature Oxidative stress (methyl viologen)	Membrane integrity, photosynthetic activities, survival rate Survival rate Electrolyte leakage Membrane integrity, photosynthetic activities Photosynthetic parameters,	Tognetti et al., 2006; Zurbriggen et al., 2008; Ceccoli et al., 2011, 2012; Giró et al., 2011
Walnut (*Juglans regia* L.)	Cyanobacterial	*Agrobacterium*-mediated	Drought & Salinity	Survival rate	Sheikh Beig Goharrizi et al., 2016

*PSII, Photosystem II; ROS, Reactive oxygen species*.

Intermediate overexpression of the *Fld* gene can also change plant physiological characteristics. Inoculation of alfalfa (*Medicago sativa*) plants with transformed bacterial cells of *Sinorhizobium meliloti* with the *Anabaena variabili Fld* gene led to a modified antioxidant metabolism, delayed nodule senescence, and improved starch accumulation in nodules (Redondo et al., 2009). Alfalfa plants nodulated by the flavodoxin-overexpressing rhizobia showed more tolerance to cadmium than plants nodulated by wild-type bacteria (Shvaleva et al., 2010).

In addition to *Fld*, other modifications of the acceptor end of PSI can affect plant stress tolerance (Gómez et al., 2019). Plant ferrodoxin-like protein (*PFLP*) and *FNR* as well as cyanobacterial flavodiiron (*Flv*) are applicable in this regard. Overexpression of *PFLP-1* and *PFLP-2* genes led to enhanced salinity tolerance in transgenic rice plants through higher antioxidant enzyme activities, higher ABA accumulation, up-regulated expression of stress-related genes (OsRBOHa, Cu/Zn SOD, OsAPX, OsNCED2, OsSOS1, OsCIPK24, OsCBL4, and OsNHX2), and lower leaf sodium ion content (Huang et al., 2020). Enhanced heat stress tolerance has been reported in *Arabidopsis thaliana* with the *PFLP* transgene (Lin et al., 2015). The chloroplastic overexpression of a pea (*Pisum sativum*) *FNR* led to increased oxidative stress tolerance in transgenic tobacco plants (Rodriguez et al., 2007). The introduction of cyanobacterial *Flv1*/*Flv3* into the tobacco chloroplasts triggered a faster recovery of plant photosynthetic performance coupled with efficient electron transport and non-photochemical quenching during dark–light transitions in transgenic plants under light intensities. These results indicate the protective role of Flvs (Gómez et al., 2018). Shahinnia et al. (2020) introduced cyanobacterium *Flv1* and *Flv3* genes to barley, using the *Agrobacterium*-mediated gene transformation method. They observed accelerated days to heading, increased biomass, promotion of the number of spikes and grains per plant, and improved grain yield of the transgenic barley plants under drought stress. Using reducing equivalents of the PETC, Flv proteins are able to direct the reduction of O_2_ to H_2_O and decrease O_2_ concentration in chloroplasts (Gómez et al., 2019).

## Roles Other Than Enhancing Stress Tolerance for *BADH* and *FLD* Transgenes

In addition to stress tolerance, *BADH* introduction has been applied for changing the fruit size and productivity of plants. Zhang T. et al. (2019) studied the effect of GB on tomato fruit development using *codA* and *BADH* transgenic lines and reported that *codA* and *BADH* transgenes led to the formation of enlarged flowers and fruits in comparison with wild-type plants. The observed fruit size enlargement was related to the contents of phytohormones, such as auxin, brassinolide, gibberellin, and cytokinin. In addition, the expression level of certain genes related to fruit growth and development was increased in transgenic lines. Transgenic and wild-type plants were mainly different in the expression level of the genes involved in photosynthesis, DNA replication, plant hormone signal transduction, and biosynthesis pathways. These results indicated that GB promoted tomato fruit development through multiple pathways, and, therefore, genetic engineering of GB synthesis can be used as a novel method to increase the productivity of tomatoes and other important plants (Zhang T. et al., 2019). *Agrobacterium*-mediated delivery of a spinach *BADH* gene changed the phytochemical profile of the ajowan medicinal plant and led to a significant increase in the thymol content of *BADH*-transformed plants compared with wild-type plants under drought stress conditions (Niazian et al., 2019). This report demonstrated the effective role of the *BADH* gene in changing the phytochemical profile of plants and its potential to improve the commercial value of medicinal and aromatic plants. *Fld* introduction can also change the plant's size and productivity as well as other biochemical processes. *Flavodoxin* expression increased the nitrogen fixation of the legume *Medicago truncatula* and therefore can be used to improve legume symbiotic performance under stressful conditions (Coba de la Peña et al., 2010). Transgenic tobacco lines expressing a plastid-targeted cyanobacterial *Fld* gene showed a decreased accumulation of ROS in their chloroplasts. *Fld* expression also delayed senescence in aging leaves by improving the maintenance of chlorophyll-protein complexes, photosynthetic electron flow, CO_2_ assimilation, central metabolic routes, and levels of bioactive cytokinins and auxins (Mayta et al., 2018). Expression of a chloroplast-targeted cyanobacterial *Fld* led to an increased harvest index in tomatoes by increasing plant density (a higher fruit number per plant in smaller plants) (Mayta et al., 2019). Thus, this report showed that *Fld* introduction had a great potential to extend flower longevity and shelf life and subsequently increase the commercial value of ornamental plants. These results implied that both *BADH* and *Fld* genes transformation had promising potential to improve major economically important traits of targeted plants in addition to enhancing stress tolerance.

## Conclusion

A rising worldwide population and food shortages due to the impact of climate change and the adverse effects of several environmental stresses on crop productivity, particularly drought and salinity, remark the importance of the rapid development of stress-tolerant plants, able to maintain sustainable productions in harsh environments. Plants employ different adaptation mechanisms, at morphological physiological, biochemical, and molecular levels, over their evolutionary course in response to challenging environmental situations. By developing transgenic plants, plant breeders are able to efficiently combat abiotic stresses. Biotechnology can enhance the tolerance mechanisms of plants by using the up and down-regulation of genes involved in plant stress responses. Genetic engineering of important compatible solutes such as GB, through overexpressing of key genes involved in their biosynthesis pathway, is a creative biotechnological approach that enhances the stress tolerance of several plant species. *BADH* is one of the key genes in the biosynthesis pathway of GB, and its introduction led to an increased accumulation of GB and subsequently an improved photosynthetic rate and performance of transgenic plants under different abiotic stresses. The introduction of effective tolerance genes from other types of living organisms is another solution to enhance plant stress tolerance through genetic engineering methods. Cyanobacterial *Fld* is one of these genes whose delivery led to increased stress tolerance in different plant species. Both *BADH* and *Fld* genes are involved in strengthening photosynthetic apparatus under hostile environmental conditions by affecting PSII and PSI, respectively. Recent studies have shown that both *BADH* and *Fld* transgenes are involved in productivity and other commercially important traits, such as larger fruit size, delayed senescence in aging leaves, as well as the content of beneficial secondary metabolites. These findings indicate the important roles of *BADH* and *Fld* transgenes and their potentials to enhance economically important characteristics of different plant species in stressful environments. The cumulative effect of *BADH* and *Fld* transgenes would be much more valuable in this regard. In conclusion, biosafety and biosecurity concerns around genetically modified organisms (GMO), and the biological safety of transgenic plants is pivotal. Therefore, the need to carefully assess the effect of GM plants on the environment, in particular on the potential issues of persistence and invasiveness, the compatibility of GM plants with related plants, gene transfer between GM and non-GM plants and microorganisms, the effects on biogeochemical processes and the effects on human and animal health, should be considered in gene transformation studies. New targeted genome editing methods can solve some public concerns about GM plants such as the integration of DNA constructs in transgenic plants, integration of two or more T-DNA copies into the plant genome, and the presence of selectable marker genes in GM plants.

## Data Availability Statement

The raw data supporting the conclusions of this article will be made available by the authors, without undue reservation.

## Author Contributions

MN conceived the idea and wrote the whole body of the manuscript. SS-N and MT supervised the project. SM was the advisor of the project. PS reviewed the manuscript. All authors contributed to the article and approved the submitted version.

## Conflict of Interest

The authors declare that the research was conducted in the absence of any commercial or financial relationships that could be construed as a potential conflict of interest.

## Abbreviations

ABA: Abscisic acid
APX: Ascorbate peroxidase
BADH: Betaine aldehyde dehydrogenase
CAT: Catalase
CMO: Choline monooxygenase
Fd: Ferredoxin
Fld: Flavodoxin
Flv: Flavodiiron
GB: Glycine betaine
GM: genetically modified
MDA: Malondialdehyde
MS: Murashige and Skoog medium
PETC: Photosynthetic electron transport chain
PFLP: Ferrodoxin-like protein
POX: Peroxidase
PSI: Photosystem I
PSII: Photosystem II
ROS: Reactive oxygen species
SOD: Superoxide dismutase.
